# 

**Published:** 1905-12

**Authors:** 


### The Library of the Bristol Medico-Chirurgical Society.

*The following donations have been received since the publication of the List in September:*

*November 30th, 1905.*

| | |
|---|---|
| J. Paul Bush, C.M.G. (1) ... ... ... ... | 4 volumes. |
| J. Michell Clarke, M.D. (2)... ... ... ... | 5 ,, |
| Nelson C. Dobson (3) ... ... ... ... | 2 ,, |
| George M. Gould, M.D. (4)... ... ... ... | 1 volume. |
| L. M. Griffiths (5) ... ... ... ... ... | 21 volumes. |
| W. Arbuthnot Lane (6) ... ... ... ... | 1 volume. |
| J. H. Loch, M.D. (7)... ... ... ... ... | 84 volumes. |
| Royal College of Physicians of London (8) ... | 1 volume. |
| R. Shingleton Smith, M.D. (9) ... ... ... | 3 volumes. |
| The Surgeon-General, United States Army (10) ... | 1 volume. |
| Council of University College, Bristol (11) ... | 1 ,, |

Unbound periodicals have been presented by Dr. Shingleton Smith, and pamphlets have been received from Mr. L. M. Griffiths.

---

### FIFTY-EIGHTH LIST OF BOOKS.

The titles of books mentioned in previous lists are not repeated.

The figures in brackets refer to the figures after the names of the donors, and show by whom the volumes were presented. The books to which no such figures are attached have either been bought from the Library Fund or received through the *Journal*.

*Atlas of Clinical Medicine, Surgery, and Pathology, An* Fasc. XXII. ... 1904

**Bain and W. Edgecombe, W.** *Harrogate Waters, Baths and Climate* ... 1905

**Ballantyne, J. W.** ... *Manual of Antenatal Pathology and Hygiene—The Embryo* ... ... ... ... ... ... ... ... ... 1904

**Barton, G. A. H.** ... *A Guide to the Administration of Ethyl Chloride* ... 1905

**Bramwell, B.** ... ... *Clinical Studies.* Vols. I.—III. ... ... ...(9) 1903-05

**Bruce, J. M.** ... ... *Materia Medica and Therapeutics.* New [7th] Ed. 1905

**Bull, S.** ... ... ... *Aneurysma Aorta* ... ... ... ... ... ... ... ... 1905

**Capern, T.** ... ... ... *Mesmeric Facts* ... ... ... ... ... ... ... (5) 1870

**Carless, W. Rose and A.** *A Manual of Surgery*... ... ... ... 6th Ed. 1905

*Catalogue (Index-) of the Library of the Surgeon-General's Office, U.S. Army,* 2nd ser., Vol. X.... ... ... ... ... ... (10) 1905

*Catalogue of Accessions to the Library of the Royal College of Physicians of London* ... ... ... ... ... ... ... ... (8) 1905

*Catalogue of the Library of the Pharmaceutical Society of Great Britain* (5) 1901

**Caton, R.** ... ... ... *How to Live* ... ... ... ... ... ... 2nd Ed. 1905

**Crichton, A.** . . ... *On Mental Derangement,* 2 vols. .. ... ... (5) 1798

**Crichton-Browne, Sir J.** *The Prevention of Senility and a Sanitary Outlook* 1905

**Dubois [—]** ... ... *Les Psychonéuroses et leur Traitement moral* (2) 1904

**Duncan, jun., A.** ... *Edinburgh New Dispensatory*... ... (5) 11th Ed. 1826

**Edgecombe, W. Bain and W.** *Harrogate Waters, Baths and Climate* ... 1905

**Godlee, R. J.** ... ... *An Atlas of Human Anatomy.* 2 vols. ... (3) [N.D.]

**Gordon, G. A.** ... ... *The Complete English Physician* (5) New Ed. [*c.* 1780]

**Gould, G. M.** ... ... *Biographic Clinics.* Vol. III. ... ... ... (4) 1905

**Hare, F.** ... ... ... *The Food Factor in Disease.* 2 vols. ... ... 1905

**Hartridge, G.** ... ... *Refraction of the Eye* ... ... ... (7) 9th Ed. 1898

**Herschell, W.** ... ... *Indigestion* ... ... ... ... ... ... 3rd Ed. 1905

**Hurst, A. M. Marshall and C. H.** *A Junior Course of Practical Zoology.* Revised by F. W. Gamble. ... ... 6th Ed. 1905

**Hutchison and H. Rainey, R.** *Clinical Methods*... ... ... ... 3rd Ed. 1905

**Kaminer, H. Senator and S.** [Eds]. *Health and Disease in Relation to Marriage.* Trans. by J. Dulberg. Vol. II. [1905]

**Lane, W. A.** .. ... *Cleft Palate and Hare Lip* ... ... ... ... (6) 1905

**Lower, R.** ... ... ... *Dr. Lower's and several other Physicians' Receipts* (5) 1700

**Luff and F. J. M. Page, A. P.** *A Manual of Chemistry* ... ... 3rd Ed. 1905

**Macdonald, D. M.** ... *Ambulance Examination Questions* ... ... ... 1905

**M'Gregor, A. N.** ... *A System of Surgical Nursing* ... ... ... ... [1905]

**McLatchie, J. D. P.** ... *Electrolysis in the Treatment of Facial Blemishes* 1905

**Marshall and C. H. Hurst, A. M.** *A Junior Course of Practical Zoology.* Revised by F. W. GAMBLE. ... ... 6th Ed. 1905
**Martindale, W. H.** ... *A Synopsis of the Principal Changes in the U. S. Pharmacopœia, 1900* ... ... ... ... ... ... [1905]
**Morris, J.** ... ... ... *Germinal Matter and the Contact Theory* (5) 2nd Ed. 1867
**Murphy, J. B.** ... ... *Tuberculosis of the Female Genitalia and Peritoneum* ... ... ... ... ... ... ... ... ... (1) 1903
**Newman, B. A. Whitelegge and G.** *Hygiene and Public Health.* New Ed. 1905
**Noorden and H. Salomon, C. von.** *Drink Restriction* ... ... .. ... 1905
**Nuttall, G. C.** ... ... *Guide to Leicester and Neighbourhood* ... ... (5) 1905
**Page, A. P. Luff and F. J. M.** *A Manual of Chemistry* ... ... 3rd Ed. 1905
*Pamphlets.* 3 vols. ... ... ... ... ... ... ... ... ... ... ... ... (5) [V.Y.]
**Paton, D. N.** ... ... *Essentials of Human Physiology* ... ... 2nd Ed. 1905
**Rainey, R. Hutchison and H.** *Clinical Methods* ... ... ... ... 3rd Ed. 1905
**Rawling, L. B.** ... *Landmarks and Surface Markings* ... 2nd Ed. 1905
**Roberts, F. T.** ... ... *The Theory and Practice of Medicine.* 2 vols. 10th Ed. 1905
**Rolleston, H. D.** ... *Diseases of the Liver, Gall-Bladder and Bile Ducts* 1905
**Rose and A. Carless, W.** *A Manual of Surgery* ... ... ... ... 6th Ed. 1905
**Russell, J. B.** ... ... *Public Health Administration in Glasgow.* Edited by A. K. CHALMERS ... ... ... ... ... ... ... 1905
**Salomon, C. von Noorden and H.** *Drink Restriction* ... ... ... ... 1905
**Sandwith, F. M.** ... *The Medical Diseases of Egypt.* Part I. ... ... 1905
**Seigneux, R. De** ... *Le Livre de la Sage-Femme et la Garde* ... ... 1905
**Senator and S. Kaminer, H.** [Eds.] *Health and Disease in Relation to Marriage.* Trans. by J. DULBERG. Vol. II. [1905]
**Shaw, H. B.** ... ... *Organotherapy* ... ... ... ... ... ... ... ... ... 1905
**Shaw-Mackenzie, J. A.** *The Nature and Treatment of Cancer.* 2nd Ed. 1905
**Stewart, W.** ... ... *Treatment of Hepatic Disease by Chloride of Ammonium* ... ... ... ... ... ... ... ... (7) 1879
**Thompson, Sir H.** ... *Diet in Relation to Age and Activity* ... ... (5) 1887
**Thomson J. A.** ... ... *Outlines of Zoology* ... ... ... ... ... ... (7) 1892
**Treves, F.** ... ... ... *Surgical Applied Anatomy* ... ... (7) 4th Ed. 1889
**Whitelegge and G. Newman, B. A.** *Hygiene and Public Health.* New Ed. 1905
**Williams, C.** ... ... *A Plea for the More Energetic Treatment of the Insane* ... ... ... ... ... ... ... ... ... 1905
**Williams, J.** ... ... *On Procuring Sleep in Insanity* ... ... ... (5) 1845
**Wood, H. C.** ... ... *Therapeutics: Its Principles and Practice.* 12th Ed. 1905

### TRANSACTIONS, REPORTS, JOURNALS, &c.

American Journal of Obstetrics, The ... ... ... ... ... Vol. LI. 1905
Boston Medical and Surgical Journal, The ... ... ... Vol. CLII. 1905
Bristol Health Report for 1904 ... ... ... ... ... ... ... .. 1905
Buffalo Medical Journal ... .. ... ... ... ... .. ... ... Vol. LX. 1905
Clinical Journal, The ... ... ... ... ... ... ... ... ... Vol. XXVI. 1905
Clinical Society's Transactions ... ... ... ... ... ... Vol. XXXVIII. 1905
Edinburgh Obstetrical Transactions ... ... ... ... Vol. XXX. 1905
Glasgow Medical Journal, The ... ... .. ... ... ... Vol. LXIII. 1905
Hospital, The ... ... ... ... ... ... ... ... ... ... Vol. XXXVIII. 1905

Hospitals—
Montreal General Hospital, Pathological Reports (2) Nos. III., IV. [1897-1903]
Statistical Tables of St. Bartholomew's Hospital, 1904 ... ... 1905
Ireland, Transactions of the Royal Academy of Medicine in. Vol. XXIII. 1905
Library World, The ... ... ... ... ... ... ... ... ... Vol. VII. 1905
Medical Chronicle, The ... ... ... ... ... ... 4th ser., Vol. IX. 1905
Medical Library and Historical Journal ... ... ... ... Vol. II. 1904
New York, Transactions of the Medical Society of the State of ... 1905
New Zealand Medical Journal ... ... ... ... N.S., Vols. II., III. 1901-04
Practitioner, The ... ... ... ... ... ... ... ... ... [Vol. LXXIV.] 1905
Progressive Medicine ... ... ... ... ... ... ... ... ... Vol. III. 1905
Sanitary Record, The ... ... ... ... ... ... ... ... Vol. XXXV. 1905
Scottish Medical and Surgical Journal, The... ... ... ... Vol. XV. 1905
University College Bristol, Calendar of ... ... ... ... ... ... (11) 1905

### MEETINGS OF SOCIETIES.

### Bristol Medico-Chirurgical Society.

### Local Medical Notes.

**University College, Bristol.**—Professor Fawcett has been appointed Dean of the Medical Faculty, *vice* Dr. E. Markham Skerritt resigned. J. Michell Clarke, M.A., M.D., F.R.C.P., has been appointed Professor of Medicine. P. Watson Williams, M.D., has been appointed Lecturer on Diseases of the Nose and Throat.

The following examination successes of students of the College have recently been recorded :—

F.R.C.S. ENG.—*Primary Examination:* C. Clarke, S. C. Hayman.

CONJOINT BOARD OF ENGLAND.—*Chemistry and Physics:* D. Y. Hylton. *Medicine:* J. H. Board,* A. J. Wright,* F. G. Bergin.* *Surgery:* H. O. Gough,* A. N. Thomas,* J. H. Board,* and H. K. Salisbury. *Midwifery:* J. S. Avery.

L.D.S.—*Final Examination:* C. H. Taylor.

* Completes Examination.

**Wrexham Infirmary.**—C. G. Russ Wood, F.R.C.S., has been appointed Honorary Ophthalmic and Aural Surgeon.

**Bristol Royal Infirmary.**—A. Rendle Short, M.D., B.S., B.Sc. (Lond.), M.R.C.S., L.R.C.P., has been appointed House Surgeon.

**Bristol Royal Hospital for Sick Children and Women.**—Mr. J. H. Lavington, M.B.(Durh.), has been appointed Assistant House Surgeon.

**Bristol Dispensary.**—Alfred Coleridge, M.B., B.S., and Arthur W. C. Richardson, M.B. Lond., were appointed Surgeons.

**Newport General Hospital.**—Mr. A. N. Thomas, M.R.C.S., L.R.C.P., has been appointed House Physician.

**Bristol Royal Infirmary.**—The half-yearly meeting of the Governors of this institution was held on September 28th, under the presidency of Sir George White. The chairman stated that there was a considerable increase in the income for the half-year ending January, 1905, as compared with the six months ended January, 1904. He alluded with satisfaction to the fact that the unfavourable balance of £15,552 against the Infirmary had been paid off, but there still remained a sum of £35,000 to be raised to bring the institution up to modern requirements.

**General Hospital.**—The half-yearly meeting of the Governors was held on September 11th, under the presidency of Mr. Proctor Baker. The chairman stated that for the first six months of 1905 the average daily number of in-patients was 179.3. The financial statement was satisfactory. Additional accommodation for the nurses and new isolation wards are required. These extensions will entail an outlay of about £12,000, and it will be necessary for the Governors to make an appeal for that sum.

**Memorials to Dr. Edward Crossman.**—The Dowager Duchess of Beaufort formally opened the new building of the Hambrook Cottage Hospital, which has been erected at a cost of about £900 as a memorial to the late Dr. Edward Crossman, who died in 1904, and who practised for many years in the district. The new building has accommodation for six patients, five in two wards on the ground floor and one in a ward on the first floor. It replaces two cottages which were first used as a hospital in 1867 on the suggestion of Dr. Crossman, who continued as medical officer of the institution until within a few years of his death. On October 21st the Bishop of Bristol dedicated two handsome frescoes which have just been placed in the sanctuary of the chapel attached to the Diocesan Training Institute at Fishponds. They represent the raising of the daughter of Jairus and Christ blessing the children, and are a memorial to the late Dr. Edward Crossman, of

Hambrook, who for over over fifty years was medical officer to the institution.

**Voluntary Notification of Pulmonary Tuberculosis.**—This practice is now being carried out in Bristol. A fee of 2s. 6d. is paid for each case notified by a private medical attendant, unless the case has been notified by another practitioner, in which case 1s. is paid. The fee paid to medical officers of public institutions is 1s. For each change of address reported, giving the new address, a fee of 1s. is paid. The Health Committee of the Bristol Town Council has arranged to secure the examination of specimens of sputum for medical men free of cost.

### CARDIFF.

**Cardiff Infirmary.**—During the past twenty-four years the expenditure of this Infirmary has exceeded the income in fifteen years. In 1903 there was a balance of £371 on the right side, but this favourable result had not occurred since 1893. In 1904 the adverse balance was £1,654, though it is only right to say that the average number of patients in the Infirmary day by day during the year was 156, as compared with 122, the average of the preceding ten years. The number of out-patients was 19,525, compared with a yearly average in the previous ten years of 13,567. At a special meeting it was decided to appoint a committee to inquire into the abuse of the out-patient department, and the best means of economising the administration of the department without impairing its efficiency.

### DEVIZES.

**Devizes Joint Isolation Hospital.**—This Hospital, recently erected jointly for the Devizes Urban and Rural District Council, was formally opened on October 31st. It contains twenty-two beds in the main building, and there is in addition a small pavilion for cases of diphtheria. The cost of the undertaking has been £10,269.

### EXETER.

**West of England Eye Infirmary.**—At the annual meeting of the Governors, held recently, the medical report stated that during the past year 3,189 patients had been treated, and that the daily average number of in-patients was 51. The financial statement was satisfactory, and showed that the Committee had been able to invest £500.

### INDEX.

(R.)=Review.

Abdominal pain—Dr. A. E. Maylard (R.), 162.
Acne vulgaris and its treatment—Dr. W. K. Wills, 113.
Adrenalin, Danger of, 252.
Albuminuria, Physiological, 239.
Alexander, Obituary notice of James, 189.
Allbutt, Dr. T. C.—Notes on the composition of scientific papers (R.), 66; The historical relations of medicine and surgery to the end of the sixteenth century (R.), 254; The prevention of apoplexy, 1.
Anæsthesia, Golden rules of—Dr. R. J. Probyn-Williams (R.), 70.
Anatomy, Applied — Dr. E. H. Taylor (R.), 255.
Anatomy, Surface—Dr. T. G. Moorhead (R.), 166; Dr. L. B. Rawling (R.), 60.
Antiseptic, The (R.), 72.
Anti-vivisection, The case against—S. Paget (R.), 72.
Anus, Diseases of the rectum and—Dr. C. H. Leaf (R.), 262.
Apoplexy, The prevention of—Dr. T. C. Allbutt, 1, 74.
Appendicitis, A study of the records of 155 cases of operation for—C. A. Morton, 223, 360.
Appendicitis, Clinical lectures on—G. R. Turner (R.), 169.
Asepsis, The principles and practice of—Dr. A. S. Vallack (R.), 265.
Assouan, Notes on—G. D. Edwards (R.), 70.

Bain, Dr. W. [Ed.]—A text-book of medical practice (R.), 257.
Baldness, The causes and treatment of—Dr. H. Waldo, 107.
Barwell, R.—Lateral curvature of the spine and pelvic deviations (R.), 169.
Begg, Dr. C.—Sprue, 138.
Bell, Dr. R.—Cancer: its causation and curability without operation (R.), 69.
Bennett, Sir W.—Recurrent effusion in the knee-joint (R.), 266.
Berry, Dr. G. A.—Manual of practical ophthalmology (R.), 172.
Bidwell, L. A.—A handbook of intestinal surgery (R.), 266.
Blair, Dr. C.—Errors of refraction and their treatment (R.), 377.
Blood pressure, 148.
Bosanquet, Dr. W. C.—Serums, vaccines and toxines (R.), 264.
Bourcart et F. Cautru, Drs. M.—Le ventre (R.), 63.
Bramwell, Dr. J. M.—Hypnotism (R.), 62.
Bristol health report (R.), 70.
Bristol Medico-Chirurgical Society, 84, 184, 395.
Bristol Royal Infirmary, 278.
British Medical Association, Leicester meeting of the, 270.
Burdett's hospitals and charities (R.), 269.
Burnet, Dr. R. W.—Foods and dietaries (R.), 166.
Burton-Fanning, Dr. F. W.—The open-air treatment of pulmonary tuberculosis (R.), 260.

Cancer, Hypodermic medication in the treatment of inoperable—Dr. J. A. Shaw-Mackenzie (R.), 171.
Cancer: its causation and curability without operation—Dr. R. Bell (R.), 69.
Carbolic acid, Nephropexy by means of the application of strong—T. Carwardine, 24.
Carpal scaphoid, Fracture of, 372.
Carr, Obituary notice of Alexander, 94.

Carwardine, T.—Nephropexy by means of the application of strong carbolic acid and temporary support, 24; Report on surgery, 244.
Catalogue (Index-) of the Library of the Surgeon-General's Office, United States Army (R.), 71.
Cautru, Drs. M. Bourcart et F.—Le ventre (R.), 63.
Cerebellar tumours, On some symptoms of—Dr. J. M. Clarke. 97.
Cerebro-spinal meningitis, 151.
Charles, Dr. J. R.—Report on medicine, 368.
Childbed, Deaths in—Dr. W. Williams (R.), 171.
Child's diet, The.—J. Sadler Curgenven (R.), 379.
Cholelithiasis, The surgical aspect of—Dr. J. Swain, 208.
Clarke, Dr. J. M.—Hysteria and neurasthenia (R.), 258; On some symptoms of cerebellar tumours, 97; The relation of medicine to the natural sciences, 193; Two cases of myasthenia gravis, 308.
Cohnheim, Dr. P.—Die krankheiten der verdauungskanal (R.), 259.
Colour and race, 387.
Colour blindness, The services and, 253.
Composition of scientific papers, Notes on the—Dr. T. C. Allbutt (R.), 66.
Consumption, Case paper and chart for (R.), 168.
Consumption, Pulmonary—Dr. A. Latham (R.), 71.
Cornea, The healing of incisions in the, 250.
Cotton, Dr. W.—Twin X-ray representation and the reflecting stereoscope, 216.
Cowen, R. J.—X-rays: their employment in cancer and other diseases (R.), 170.
Cunning, Dr. J.—Aids to surgery (R.), 169.
Curgenven, J. S.—The child's diet (R.), 379.

Dacre, J.—The family doctor, 289.
Davies, Dr. D. S.—The voluntary notification of phthisis, 351.
Davis, O. C. M.—The progress of pharmacy and pharmaceutical chemistry, 120.
Dermatology, An introduction to—Dr. N. Walker (R.), 268.

Ear, Diseases of the—Dr. J. K. Love (R.), 261; R. Lake (R.), 69.
Ears, The rational treatment of running.—F. F. White (R.), 378.
Edgeworth, Dr. F. H.—On some anomalous cases of locomotor ataxy, 234.
Edinburgh obstetrical transactions (R.), 172.
Editorial notes, 73, 173, 270, 380.
Edwards, G. D.—Notes on Assouan (R.), 70; Obituary notice of, 398.
Electricity, Medical—Dr. H. L. Jones (R.), 265.
Experiments on animals, What we owe to—S. Paget (R.), 72.
Eyeball, Excision of the, 249.

Family doctor, The.—J. Dacre, 289.
Fernie, Dr. W. T.—Meals medicinal (R.), 64.
Firth, Dr. J. L.—Report on surgery, 53.
Fisher, Dr. J. H.—Ophthalmological anatomy (R.), 63.
Fisher, Dr. T.—Report on medicine, 49.
Foods and dietaries—Dr. R. W. Burnet (R.), 166.
Fortescue-Brickdale, Dr. J. M.—A new scheme for infant milk depôts, 132; A note on congenital dilatation of the ureters with hydronephrosis, 231.
Fox lecture, The Long, 317.
Froussard, Dr. P.—Muco-membranous enterocolitis (R.), 167.

Gant, Autobiography of Frederick James (R.), 165.
Gastric ulcer, Surgical treatment of hæmatemesis from, 371.
Graves's disease, The pathology and treatment of—Dr. R. S. Smith, 317
Griffiths, L. M.—Medical philology (R.), 165.
Griffiths, Obituary notice of Arthur David, 284.
Grimsdale, Dr. H. B.—Errors of refraction and their correction (R.), 268; The chief operations of ophthalmic surgery (R.), 64.

Groves, Dr. E. W. H.—On the advantages of enucleation of the tonsils over their removal by the guillotine, 32.

Hæmatemesis from gastric ulcer, Surgical treatment of, 371.
Hall and G. Herxheimer, Drs. I. W. —Methods of morbid histology and clinical pathology (R.), 256.
Heart, Arhythmia of the—Dr. K. F. Wenckebach (R.), 61.
Herxheimer, Drs. I. W. Hall and G.—Methods of morbid histology and clinical pathology (R.), 256.
Higgens, C.—A manual of ophthalmic practice (R.), 267.
Histology and clinical pathology, Methods of morbid—Drs. I. W. Hall and G. Herxheimer (R.), 256.
Historical relations of medicine and surgery—Dr. T. C. Allbutt (R.), 254.
Hospital abuse, 75.
Hospitals and charities, Burdett's (R.), 269.
Hydronephrosis, Congenital dilatation of the ureters with — Dr. J. M. Fortescue-Brickdale, 231.
Hypnotism — Dr. J. M. Bramwell (R.), 62.
Hysteria and neurasthenia — Dr. J. M. Clarke (R.), 258.

Inebriety and the so-called cures— J. Stewart, 144.
Infant life, 276.
Infant milk depôts — Dr. J. M. Fortescue-Brickdale, 132.
Insanity, A short essay on — C. Williams (R.), 66.
Intestinal surgery—L. A. Bidwell (R.), 266.

Jaundice, Benign infectious, 243.
Joint swellings simulating gout and rheumatism—Dr. R. L. Jones 127.
Jones, Dr. H. L.—Medical electricity (R.), 265.
Jones, Dr. R. L.—Cases of joint swellings simulating gout and rheumatism, 127.
Journal of the Röntgen Society, The (R.), 70.

Keratitis in the new-born, Traumatic, 251.
Keratitis, Interstitial, 252.
Knee-joint, Recurrent effusion in the—Sir W. Bennett (R.), 266.

Lack, Dr. L. A. H.—An introduction to physiology (R.), 375.
Lake, R.—Handbook of diseases of the ear (R.), 69; Laryngeal phthisis (R.), 377.
Lamb, Dr. W.—Guide to the examination of the throat, nose, and ear (R.), 268.
Landmarks and surface anatomy— Dr. L. B. Rawling (R.), 60.
Laryngeal phthisis.—R. Lake (R.), 377.
Laryngology, Report on—Dr. P. W. Williams, 57.
Larynx, Malignant disease of the— Dr. P. R. W. De Santi (R.), 65.
Latham, Dr. A. —The diagnosis and modern treatment of pulmonary consumption (R.), 71.
Leaf, Dr. C. H.—Surgical case book charts for rectal diseases (R.), 262; The diagnosis and treatment of some of the common diseases of the rectum and anus (R.), 262.
Library of the Bristol Medico-Chirurgical Society, 82, 181, 281, 392.
Life assurance, Some notes on examinations for — Dr. R. S. Smith, 11.
Light treatment for nurses, Treatise on—Dr. J. H. Sequeira (R.), 171.
Local medical notes, 95, 190, 285, 398.
Locomotor ataxy, On some anomalous cases of — Dr. F. H. Edgeworth, 234.
London, Transactions of the Medical Society of (R.), 168.
London, Transactions of the Obstetrical Society of (R.), 172.
Love, Dr. J. K.—Diseases of the ear (R.), 261.

McKay, Dr. W. J. S. — The preparation and after-treatment of section cases (R.), 162.
Maclean, Dr. E. J. — Three cases of ovarian tumour complicating pregnancy, labour and the puerperium respectively, 40.

Macnaughton - Jones, Dr. H. — Practical manual of diseases of women and uterine therapeutics (R.), 269.
Maylard, Dr. A. E. — Abdominal pain (R.), 162.
Meals medicinal—Dr. W. T. Fernie (R.), 64.
Medical annual, The (R.), 264.
Medical practice, A text-book of—Dr. W. Bain [Ed.] (R.), 257.
Medicine, Reports on—Dr. J. R. Charles, 368; Dr. T. Fisher, 49; Dr. N. Neild, 239; Dr. G. Parker, 148.
Medicine to the natural sciences, The relation of—Dr. J. M. Clarke, 193.
Milk depôts, Infant — Dr. J. M. Fortescue-Brickdale, 132.
Moorhead, Dr. T. G. — Surface anatomy (R.), 166.
Morton, C. A.—A study of the records of 155 cases of operation for appendicitis, 223, 360; Report on surgery, 153.
Moullin, Dr. C. M.—Enlargement of the prostate (R.), 71.
Muco-membranous enterocolitis—Dr. P. Froussard (R.), 167.
Mucous membranes—W. Stuart-Low (R.), 378
Myasthenia gravis, Two cases of—Dr. J. M. Clarke, 308

Nasal septal deviations by sub-mucous resection, Treatment of, 59.
Natural sciences, The relation of medicine to the—Dr. J. M. Clarke, 193.
Nauheim treatment of chronic heart disease, The—Dr. L. T. Thorne (R.), 71.
Neild, Dr. N.—Report on medicine, 239.
Nephropexy by means of the application of strong carbolic acid and temporary support — T. Carwardine, 24.
Neurasthenia, Hysteria and — Dr. J. M. Clarke (R.), 258.
New York, Transactions of the Medical Society of the State of (R.), 67.

Obituary, 92, 189, 284, 398.
Obstetrical Society of London, Transactions of the (R.), 172
Obstetrics, Report on—Dr. W. C. Swayne, 156.
Ocular circulation, The—Dr. J. H. Parsons (R.), 267.
Open-air treatment of pulmonary tuberculosis, The — Dr. F. W. Burton-Fanning (R.), 260.
Ophthalmic practice, A manual of —C. Higgins (R.), 267.
Ophthalmic surgery — Dr. H. B. Grimsdale (R.), 64.
Ophthalmological anatomy — Dr. J. H. Fisher (R.), 63.
Ophthalmology, Practical — Dr. G. A. Berry (R.), 172.
Ophthalmology, Report on — Dr. C. H. Walker, 249.
Organotherapy—Dr. H. B. Shaw (R.), 375
Ovarian tumour complicating pregnancy, Three cases of—Dr. E. J. Maclean, 40.

Paget, S.—The case against antivivisection (R.), 72; What we owe to experiments on animals (R.), 72.
Paratyphoid fever, 368.
Parker, Dr. G.—Report on medicine, 148.
Parsons, Dr. J. H.—The ocular circulation (R.), 267.
Parsons, Obituary notice of James St. John Gage, 285.
Pathology, Methods of morbid histology and clinical — Drs. I. W. Hall and G. Herxheimer (R.), 256.
Paton, Dr. D. N.—Essentials of human physiology (R.), 376.
Penny, Obituary notice of William John, 92.
Pericarditis, 49.
Pericarditis, Paracentesis of, 51, 52.
Pharmacy and pharmaceutical chemistry, The progress of — O. C. M. Davis, 120.
Philology, Medical—L. M. Griffiths (R.), 165.
Phthisis, Laryngeal—R. Lake (R.), 377.
Phthisis, The voluntary notification of—Dr. D. S. Davies, 351.

Physical education and improvement, Proposed national league for, 385.
Physiology, An introduction to—Dr. L. A. H. Lack (R.), 375.
Physiology, Essentials of human—Dr. D. N. Paton (R.), 376.
Physiology, Exercises in practical—Dr. A. D. Waller (R.), 263.
Probyn - Williams, Dr. R. J. — Golden rules of anæsthesia (R.), 70.
Prostate, Carcinoma of the, 374.
Prostate, Enlargement of the—Dr. C. M. Moullin (R.), 71.
Prostate gland, Development and anatomy of the — Dr. W. G. Richardson (R.), 256.
Prostatectomy, 244.
Puerperal sepsis, 156.

Rawling, Dr. L. B. — Landmarks and surface markings of the human body (R.), 60.
Rectal charts—Dr. C. H. Leaf (R.), 262.
Rectum and anus, Diseases of the—Dr. C. H. Leaf (R.), 262.
Reed, Dr. B.—Lectures on diseases of the stomach and intestines (R.), 161.
Refraction, Errors of—Dr. C. Blair (R.), 377; Dr. H. B. Grimsdale (R.), 268.
Rheumatic diseases, The—Dr. J. O. Symes (R.), 163.
Rhinology, Report on—Dr. P. W. Williams, 57.
Richardson, Dr. W. G.—On the development and anatomy of the prostate gland (R.), 256.
Rockefeller Institute for Medical Research, Studies from the (R.), 67.
Röntgen rays. *See* X-rays.

St. Bartholomew's Hospital reports (R.), 167.
St. Thomas's Hospital reports (R.), 168.
Sanatorium treatment of phthisis, 173.
Santi, Dr. P. R. W. De—Malignant disease of the larynx (R.), 65.
Sclerotic, Trans-illumination of the, 250.
Section cases, The preparation and after-treatment of—Dr. W. J. S. McKay (R.), 162.
Semi-lunar bone, Dislocation of, 372.
Sequeira, Dr. J. H.—An elementary treatise on the light treatment for nurses (R.), 171.
Serums, vaccines and toxines—Dr. W. C. Bosanquet (R.), 264.
Services and colour blindness, The, 253.
Shaw, Dr. H. B.—Organotherapy (R.), 375.
Shaw-Mackenzie, Dr. J. A.—Some methods of hypodermic medication in the treatment of inoperable cancer (R.), 171.
Shock, Surgical, 153.
Sick, Preparations for the, 76, 177, 278, 387.
Smith, Dr. R. S.—Some notes on examinations for life assurance, 11; The Long Fox lecture, 317.
Smithsonian Institution, Annual report of the (R.), 165.
Society, Bristol Medico-Chirurgical, 84, 184, 395.
Spine, Lateral curvature of the—R. Barwell (R.), 169.
Sprue—Dr. C. Begg, 138.
Stewart, J.—Inebriety and the so-called cures, 144.
Stomach and intestines, Diseases of the—Dr. B. Reed (R.), 161.
Stuart-Low, W. — Mucous membranes, normal and abnormal (R.), 378.
Surgery, Aids to—Dr. J. Cunning (R.), 169.
Surgery, Reports on—T. Carwardine, 244; Dr. J. Lacy Firth, 53; C. A. Morton, 153; Dr. J. Swain, 371.
Surgical operations, Student's handbook of—Sir F. Treves (R.), 69.
Surgical pathology—W. J. Walsham (R.), 265.
Swain, Dr. J.—Report on surgery, 371; The surgical aspect of cholelithiasis, 208.
Swayne, Dr. W. C. — Report on obstetrics, 156.
Symes, Dr. J. O.—The rheumatic diseases (R.), 163.

Taylor, Dr. E. H.—Applied anatomy (R.), 255.

Thoracic duct, Wounds of, 53.
Thorne, Dr. L. T.—The Nauheim treatment of chronic diseases of the heart (R.), 71.
Throat, nose, and ear, Examination of the—Dr. W. Lamb (R.), 268.
Tonsillitis due to suppurative mammitis in cows, 58.
Tonsils, Advantages of enucleation of—Dr. E. W. H. Groves, 32.
Treves, Sir F.—The student's handbook of surgical operations (R.), 69.
Tuberculin redivivus, 176.
Tuberculosis and immunity, 384.
Tuberculosis, The open-air treatment of pulmonary—Dr. F. W. Burton-Fanning (R.), 260.
Turner, G. R.—Clinical lectures on appendicitis, radical cure of inguinal hernia and perforating gastric ulcer (R.), 169.
Typhoid fever in U.S. military camps during the Spanish War of 1898, Report on the origin and spread of (R.), 263.

University College, Bristol, Faculty of Medicine, 380.
University College Colston Society, 73.
Ureters, Congenital dilatation of the—Dr. J. M. Fortescue-Brickdale, 231.
Urine, Incontinence of, 56.

Vaginal tumours—W. R. Williams (R.), 261.
Vallack, Dr. A. S.—The principles and practice of asepsis (R.), 265.
Ventre, Le—Drs. M. Bourcart et F. Cautru (R.), 63.
Verdauungskanal, Die krankheiten des—Dr. P. Cohnheim (R.), 259.
Vincent's angina, 57.

Waldo, Dr. H.—The causes and treatment of baldness, 107.
Walker, Dr. C. H.—Report on ophthalmology, 249.
Walker, Dr. N.—An introduction to dermatology (R.), 268.
Waller, Dr. A. D.—Exercises in practical physiology (R.), 263.
Walsham, [W. J.]—Handbook of surgical pathology (R.), 265.
Warner, Obituary notice of E. H., 189.
Wenckebach, Dr. K. F.—Arhythmia of the heart (R.), 61.
White, F. F.—The rational treatment of running ears (R.), 378.
Williams, C.—A short essay on insanity( R.), 66.
Williams, Dr. P. W.—Report on laryngology and rhinology, 57.
Williams, Dr. W.—Deaths in childbed (R.), 171.
Williams, Obituary notice of Eubulus, 94.
Williams, W. R.—Vaginal tumours, with special reference to cancer and sarcoma (R.), 261.
Wills, Dr. W. K.—Acne vulgaris and its treatment, 113.
Winsley sanatorium, The, 96.
Women, Diseases of—Dr. H. Macnaughton-Jones (R.), 269.

X-ray representation and the reflecting stereoscope, Twin—Dr. W. Cotton, 216.
X-rays: their employment in cancer and other diseases—R. J. Cowen (R.), 170.

J. W. Arrowsmith Printer Quay Street, Bristol.

